# Changes in Pomological and Physical Parameters in Rosehips during Ripening

**DOI:** 10.3390/plants12061314

**Published:** 2023-03-14

**Authors:** Brigita Medveckienė, Dovilė Levickienė, Nijolė Vaitkevičienė, Viktorija Vaštakaitė-Kairienė, Jurgita Kulaitienė

**Affiliations:** Department of Plant Biology and Food Sciences, Agriculture Academy, Vytautas Magnus University, Donelaicio St. 58, 44248 Kaunas, Lithuania

**Keywords:** rosehip, ripening stage, pomological parameters

## Abstract

Rosehips of various *Rosa* spp. are well known for having human health-promoting compounds like mineral nutrients, vitamins, fatty acids, and phenolic compounds. However, little is known about rosehip characteristics which describe the fruit quality and may indicate appropriate harvest times. Our study evaluated the pomological (width, length, and weight of fruits, flesh weight, and seed weight), texture, and CIE colour parameters (L*, a*, and b*), chroma (C), and hue angle (h°) of rosehip fruits of *Rosa canina*, *Rosa rugosa*, and genotypes of *Rosa rugosa* ‘Rubra’ and ‘Alba’, harvested at five ripening stages (I–V). The main results revealed that genotype and ripening stage significantly affected parameters. The significantly longest (*R. canina*) and widest fruits (*R. Rugosa*) were measured at ripening stage V. Genotypes of *R. rugosa* ‘Rubra’ and ‘Alba’ had significantly higher fruit and flesh weights at ripening stage V. Rosehips of all investigated genotypes expressed darkness (lower L*) during ripening, and had the highest hue angle h° values at ripening stage I while the lowest was at stage V. The significantly lowest skin elasticity of rosehips was found at stage V. However, *R. canina* was distinguished by the highest fruit skin elasticity and strength. As our results show, the desired pomological, colour, and texture features of various species and cultivars rosehips can be optimised according to the harvest time.

## 1. Introduction

With a growing global population and diminishing natural resources, food supply and demand disparity have become more pronounced [1]. Diverse species employed in agricultural systems are crucial for human nutrition and sustainable food systems. Food diversity gained from or near agricultural fields and natural environments such as forests is an additional source of food system resilience [2]. The hunt for nutrient-dense food sources is a matter of great concern in today’s food-scarce, multibillion-person world. An unexploited functional food source is the *Rosa* species cultivated for the ornamental flowers where the fleshy fruits (hypanthium/haw), situated under the perianth, known as rosehips, are removed [3].

As interest in rosehips’ nutritional worth has increased, many studies on their morphological traits, phytochemical components, antioxidant capacity, and volatile chemicals have been conducted [1]. In addition, in natural growing conditions, different species and varieties show diversity in most morphological traits [4]. These plants are widely utilised in food; however, the interest in using them in cosmetics and pharmaceutical industries as effective materials for enhancing the quality of final goods with antioxidant activity features is prevailing [5,6,7]. Rosehips have antioxidant and anti-inflammatory, antibacterial, anti-mutagenic probiotic, anti-ulcerogenic, antinociceptive, and anti-carcinogenic properties [8,9]. In a recent study, over 500 s metabolites rosehips from five species were determined, most of which were flavonoids and phenolic acids [1]. In addition, rosehips are a rich source of mineral nutrients [10] and vitamins (particularly, vitamin C) and are abundant in carotenoids, tocopherols, tannins, organic acids, amino acids, and pectin [11,12]. In their seeds prevail polyunsaturated fatty acids, followed by monounsaturated and saturated fatty acids [13]. Linoleic, -linolenic, and oleic fatty acids were the most abundant unsaturated fatty acids in rosehip seed oil [14]. Due to these reasons, rosehips are used in developing new products in the food and pharmacy industries [4,15,16,17]. For example, the use of rosehip meal in eggs enriched with polyunsaturated fatty acids had a positive effect on the amino acid and fatty acid content, as well as the antioxidant capacity, and was very effective in extending the shelf-life of eggs [16]. The supplementation of *Rosa canina* rosehips may be recommended as a natural colourant in poultry diets in conventional or organic egg production [17].

Wild fruits exhibit diverse morphology, fruit quality, yield, and phytochemicals compared to cultivated ones, and all those traits can be influenced by ecotypes [4]. *Rosa* spp. are native to cold and highland areas but demonstrate high environmental adaptability, as they are widespread plants in different climate regions such as Turkey [4,18,19,20], Slovakia [21], Serbia [14,22,23], Croatia [24,25], Romania [26], or Lithuania [10,12,13]. 

Several studies showed that organic plants indicate a higher nutritional value and higher content of biologically active compounds in various crops from organic compared to conventional farming [27,28]. For example, organically cultivated rosehips had significantly higher phenolic compounds and antioxidant capacity values than conventional fruits [29,30]. However, genotype seems to be the more influential factor than cultivation techniques since the content of bioactive compounds like carotenoids was higher in wild rosehips than those cultivated in the organic or conventional systems [30].

Characterising the best harvesting time is also important in improving the quality and nutritional value of the processed product. Several studies reported that the qualitative and quantitative composition of the biologically active compounds in rosehips, such as fatty acids [13], phenolic compounds, or vitamin C [12,31,32] varies during the stages of maturity. These results may lead to the assumption that the accumulation of phytochemicals may correspond to pomological or organoleptic traits, such as colour characteristics. For example, darker rosehips showed higher phytochemical concentrations and antioxidant activity [1]. Additionally, in numerous fruit crops, colour is a significant determinant of the fruit’s appearance and maturity. Typically, the harvesting period is defined by colour changes of the rosehip’s skin, which turns from green to light orange or pink into red-orange or deeper red. In the study by Uggla et al. [33], the association between colour as assessed by CIE (L*, a*, b*) coordinates and °Brix values and qualitative factors during the ripening period in *Rosa dumalis* and *R. rubiginosa* have been published. However, compared to the phytochemical composition, pomological and colour parameters have been poorly presented for other *Rosa* species, and the studies on those characteristics associated with ripening are very limited. In addition, these data are relevant for the evaluation of the harvest time of rosehips and can be used to choose cultivars for future production in Lithuanian climatic conditions.

This study was aimed at evaluating the pomological, texture, and colour parameters of the fruits of different *Rosa* species/cultivars (*R. canina*, *R. rugosa*, *R. rugosa* ‘Rubra’, *R. rugosa* ‘Alba’) harvested at five ripening stages.

## 2. Results and Discussion

### 2.1. Pomological Parameters of Rosehip Fruits

Horticulture is primarily concerned with the growth of plant material for human consumption, medicinal usage, or functional and aesthetic objectives [34]. In this context, *Rosa* spp. are important plants for traditional pharmacological practices and landscape studies; the fruit quality is assessed by many pomological parameters, which can differ with climate conditions, geographical location, or geographic ecological origin. According to a two-way ANOVA, the examined pomological characteristics of rosehip fruits were affected by species and/or cultivars, ripening stage, and their interaction (Table 1). 

Our results showed that the significantly longest fruits (24.93 mm) were those of *Rosa canina*, while the widest fruits (31.88 mm) corresponded to *Rosa rugosa* at ripening stage V. *Rosa rugosa* ‘Rubra’ and *Rosa rugosa* ‘Alba’ had significantly higher fruit weights (12.53 and 13.79 g, respectively) and flesh weights (11.33 and 12.65 mm, respectively) at ripening stage V than the other genotypes (Table 1). 

For each species and cultivar of *Rosa* spp., the weight of seeds varied from 0.39 to 1.42 g, depending on the ripening stage. *Rosa canina* had significantly greater seed weights at ripening stage V, while *R. rugosa* had significantly lower seed weights at ripening stage I. Our results agree with those of previously published studies that referred to pomological characteristics, such as fruit length, width, weight, flesh weight, seed weight, and the fruit–flesh ratio [26,35,36].

**Figure 1:**
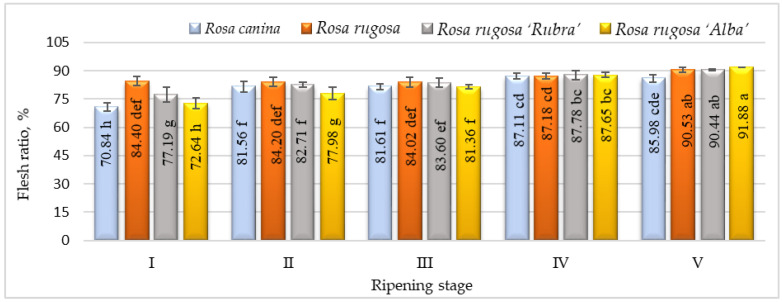
Flesh ratio of rosehip of different species/cultivars at five ripening stages (n = 60). Data are given as the arithmetic mean ± standard deviation. Averages followed by different letters are significantly different at the 5% level of probability (*p* < 0.05). The effects of species/cultivars, ripening stage, and their interaction are significant at *p* < 0.0001.

The flesh ratio is one of the most important criteria regarding rosehip fruit quality. In developing rosehip cultivars suitable for the industry, cultivars with large-sized fruits and a high flesh ratio are desired [19]. Our results indicated that the flesh ratio was quite variable, ranging from 70.84 % (ripening stage I, *R. canina*) to 91.88 % (ripening stage V, *R. rugosa* ‘Alba’) (Figure 1). This ratio was highest in *R. rugosa*, *R. rugosa* ‘Rubra’, and *R. rugosa* ‘Alba’ in ripening stage V. Previous research has also found a significant level of flesh ratio variability within *Rosa* L. species. Our results were comparable with the other studies [18,19,36,37], showing flesh ratios of 46.8–100% among rosehip genotypes. 

### 2.2. Colour Parameters of Rosehips

CIE colour parameters were determined and are shown in Table 2: L* for the value for lightness, a* value for red/green colour, b* value for yellow/blue colour, hue angle (h°), and C* for the brightness of different genotypes of rosehip fruits at each ripening stage. A two-way ANOVA showed that rosehip species/cultivar, ripening stage, and their interaction significantly influenced the CIE colour coordinates L*, a*, b*, C, h° values. According to Kazankaya et al. [37], rosehip fruits had a base colour of red, dark red, light red, and orange. Our results showed that the value of L* varied differently in all species and/or cultivars during the ripening period (Table 2). Erogul and Oguz [38] researchers established the L* value of the investigated rosehip fruits from 32.35 to 37.62 among all genotypes. According to these authors, the differences can be caused by environmental conditions and depend on genotype. Our data showed that the fruits of *R. canina*, *R. rugosa*, *R. rugosa* ‘Rubra’, and *R. rugosa* ‘Alba’ expressed darkness during ripening, as demonstrated by the decreasing values of coordinate L*. Significantly higher rosehip lightness values L* were found for *R. rugosa* at ripening stages I, II, and IV (38.44, 39.24, and 38.07, respectively) and for *R. rugosa* cv. ‘Alba’ at ripening stages I, II, and III (39.31, 38.56, and 38.17, respectively). A significantly lowest coordinate L* (26.60) was found in the fruits of *R. canina* at ripening stage V. A study by Ercisli [20] showed higher colour L* (48.06), a* (41.70), and b* (39.39) values of *R. canina* rosehip fruits. Shades of red colours were found to be predominant among the investigated genotypes. Other studies showed that the L* value varied from 30.5 to 45.2 in the rosehips harvested from four Sicilian plants of *Rosa canina* [7].

For all investigated *Rosa* spp., colour changes during ripening were characterised by a significant increase of a* (redness) (Table 2). All investigated samples exhibited negative values of a* ranging from −1.89 to −4.52 at ripening stages I and II.

All tested rosehip samples were significantly greener at ripening stage I than those harvested at other ripening stages. However, from ripening stages III to V, there was a significant increase in the a* value, changing from negative to positive values (6.96–37.83) in all rosehips. According to the literature, colour variation in fruits is due to changes in the accumulation of pigments like carotenoids and chlorophyll degradation [39]. At ripening stage V, comparing the species and/or cultivars, the redness of rosehip fruits differed. *Rosa rugosa* showed a significantly higher a* value (37.83), followed by *R. rugosa* ’Rubra’ (35.59), *R. rugosa* (35.00), and *R. rugosa* ‘Alba’ (32.25). Nine *Rosa* spp. genotypes were investigated by Bilgin et al. [40] and they found a* values from 19.31 to 34.09, which express the red colour, while the L* value was from 21.71 to 36.01 and the b* from 9.75 to 22.57.

The value of coordinate b* showed a high level of variation (Table 2). The colour b* value of fruits was always positive (yellow h°) for all species/cultivars at all ripening stages. Significantly, the highest value of coordinate b* (41.51) was found for *R. rugosa* fruits at ripening stage I and the lowest (22.53) for *R. canina* at stage V, indicating that they became less yellow. In Palermo, Fascella et al. [7] investigated colour parameters for different Sicilian rose species with intense red colour fruits. The colour b* value varied from 14.1 to 26.0. According to these researchers, the rosehip colour may be a valuable predictor of the optimal harvesting time for different *Rosa* spp. genotypes [7].

Ripening stage and genotype significantly affected the C* values of the rosehips (Table 2). Our data showed that, as the fruit ripened, the C* values of all species and (or) cultivars increased significantly. For example, in the fruits of *R. rugosa*, the value of C* was significantly higher at ripening stages IV and V (49.87 and 48.28, respectively). According to Erogul and Oguz [39], the range of the C* value varied from 34.53 to 42.25, which is similar to ours.

In all investigated rosehip fruits, the hue angle (h°) of all ripening stages was significantly different (Table 2). All tested rosehip samples had the highest h° values at ripening stage I and the lowest at stage V. *R. canina* fruits had the significantly highest h° value of 98.39 (green) and the lowest h° value of 32.77 (red). Chae et al. [31] showed that the h° of *R. rugosa* rosehips also depended on the ripeness stage. h° decreased during ripening due to carotenoid accumulation, which is associated with changes in yellow and red colours [33,41].

### 2.3. Texture Properties of Rosehip Fruits

**Figure 2:**
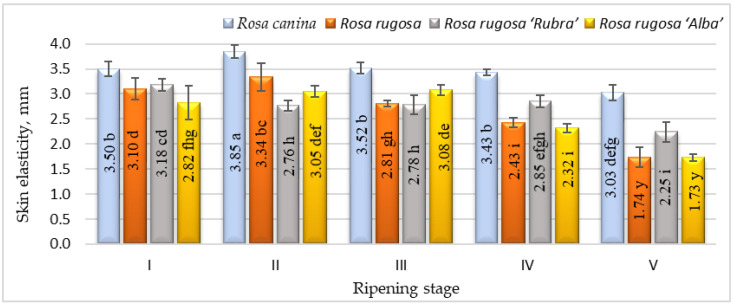
Elasticity of different species/cultivars at five ripening stages (n = 60). Data are given as the arithmetic mean ± standard deviation. Averages followed by the different letters are significantly different at the 5% level of probability (*p* < 0.05). The effects of species/cultivars, ripening stage, and their interaction are significant at *p* < 0.0001.

Ripening is the process by which fruits acquire desirable flavour, quality, colour, palatability, and other textural attributes. However, the textural aspects of the skin of rosehips during ripening have received little attention in the literature. The changes in elasticity during rosehip ripening for different cultivars and/or species are presented in Figure 2. 

**Figure 3:**
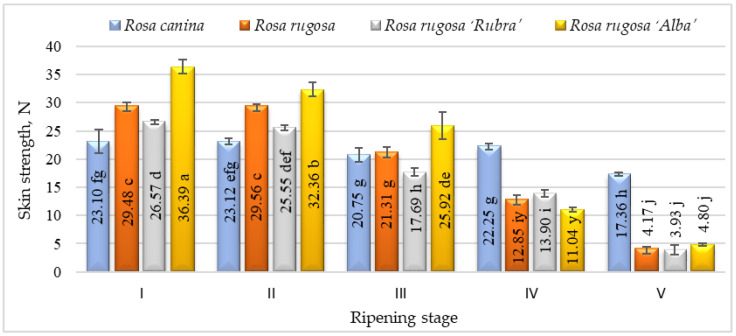
Strength of different species/cultivars at ripening stages (n = 60). Averages in the column followed by different letters are significantly different at the 5% probability level (*p* < 0.05). The effects of species/cultivars, ripening stage, and their interaction are significant at *p* < 0.0001.

The elasticity of the rosehip skin had a tendency to decrease during the ripening period and the significantly lowest elasticity was found at stage V. According to the results, *Rosa canina* presented the highest skin elasticity during ripening. We found significant differences in strength among the cultivars and/or species studied and ripening stages (Figure 3). Fruits harvested at stages I and II have shown higher skin strength than at other ripening stages. During ripening, the fruit of all investigated cultivars/species had a decrease in skin strength. The strength parameters of all species and/or cultivars also changed throughout ripening. At ripening stage V, *Rosa canina* fruit had the strongest skin compared with other genotypes at the same stage. A decrease in the strength of the fruit skin during ripening can be associated with the metabolic pathways that are responsible for textural changes in fruits, which are believed to involve loss in turgor pressure, degradation, and other physiological changes in the composition of membranes, degradation of starch, and modifications in the cell wall structure and dynamics [42].

During fruit ripening, various biochemical changes occur, including seed maturation, colour change, texture softening, volatile taste generation, wax formation on the skin, tissue permeability, and changes in the content of carbohydrates, organic acids, and proteins. These ripening-related changes account for fruit skin elasticity and strength variations during fruit maturity 28]. Unfortunately, as far as we know, there is no research on rosehip fruit skins.

### 2.4. Correlation among Investigated Traits 

The correlation matrix between the pomological, colour, and textural properties of rose hip species/cultivars is shown in Table 3. Strong positive correlations were found between fruit weight and flesh weight, seed weight, and fruit length (*r* = 0.999, *r* = 0.778, and *r* = 0.856, respectively). According to other researchers, the highest correlation was that between fruit weight and fruit width (*r* = 0.898) [40]. 

Our study showed that fruit length had a negative correlation with L ∗, b*, and h° values (*r* = −0.751, *r* = −0.641, and *r* = −0.933, respectively) and a high positive correlation (*r* = 0.910) with the a* value. In comparison, values of h° were strongly negative for a* and C (*r* = −0.933 and *r* = −0.823, respectively). Bilgin et al. [40] discovered highly negative associations between fruit weight, stone weight, and width and colour L*, a*, b*, and C* values. In our study, skin elasticity negatively correlated with fruit weight, flesh weight, and fruit width (*r* = −0.737, *r* = −0.741, and *r* = −0.876, respectively). As well, fruit skin strength correlated negatively with fruit weight, flesh weight, fruit length, and a * (*r* = −0.878, *r* = −0.880, *r* = −0.800, and *r* = −0.855) while it positively correlated with the h° value (*r* = 0.839). No correlation was found between skin elasticity, C, and rosehip L* and b* values.

### 2.5. Principal Component Analysis

**Figure 4:**
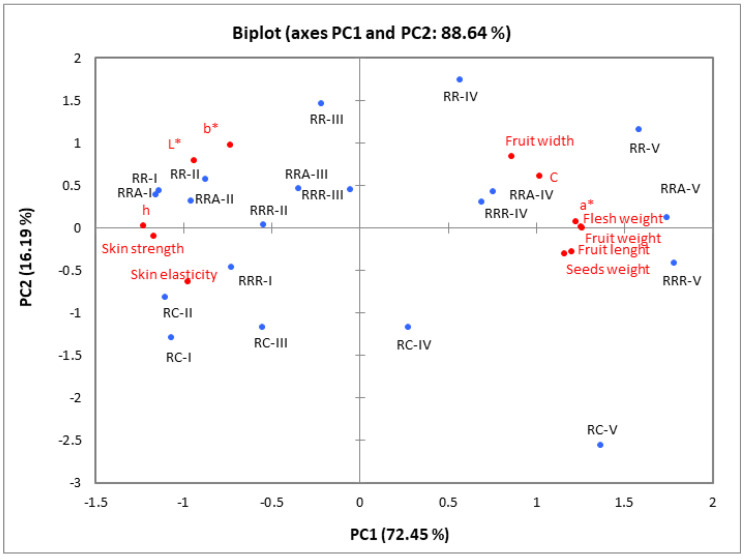
PCA for pomological (fruit weight, flesh weight, seeds weight, fruit width, fruit length), colour (L*, a*, b*, C* and h◦), and texture (skin elasticity, skin strength) parameters of the rosehip of different species and (or) cultivars. RC—*Rosa canina*, RR—*Rosa rugosa*, RRR—*Rosa rugosa* ‘Rubra’, RRA—*Rosa rugosa* ‘Alba’ harvested at five ripening stages (I, II, III, IV, and V).

The relationships between rosehip samples of different species/cultivars harvested at five ripening stages and the pomological (Table 1), colour (Table 2), and textural (Figure 2 and Figure 3) properties were examined using a principal component analysis (PCA). The PCA findings showed that the first two axes (PC1: 72.45% and PC2: 16.19%) explained 88.64% of the total variance (Figure 4). In addition, the first principal component’s (PC1) and second principal component’s (PC2) eigenvalues were both greater than one (8.69 and 1.94, respectively).

All pomological characteristics and colour characteristics a* and C were strongly and positively associated with PC1. In contrast, the skin’s colour parameters L*, h◦, and textural properties (strength and elasticity) were negatively associated with PC1. PC2 was positively associated with colour coordinate b*. As illustrated in Figure 4, PC1 separated the rosehip samples harvested at ripening stages I, II, and III from the rosehip samples harvested at ripening stages IV and V. The fruits harvested at ripening stages IV and V are defined by higher width, length and weight, and also, greater values of colour characteristics a* and C. However, these samples contained lower values of colour parameters L*, h°, skin strength, and elasticity compared to fruits harvested at ripening stages I, II, and III.

## 3. Materials and Methods

### 3.1. Field Experiments

This research was conducted in 2018–2020 at the organic farm in Pakruojis District, Lithuania (field coordinates 56°10′29.0″ N 23°49′02.6″ E). Two species, *Rosa rugosa* and *Rosa canina* and two cultivars, *Rosa rugosa* cv. ‘Rubra’ and *Rosa rugosa* cv. ‘Alba’ were planted in 2011. The distance between the rows was 4 m, and the distance between rosehip shrubs was 2 m. The interrow was loosened, and weeds were manually removed. The soil pH at the experimental site was from 6.8 to 7.0 mg kg^−1^ plant-available potassium from 97.7 to 181.0 mg kg^−1^, available phosphorus from 120.6 to 153.3 mg kg^−1^, and total nitrogen from 2.5%. The experimental plots were arranged in the randomised block with four replicates per treatment. Each replication consisted of four plants of each species/cultivar. Fruits were randomly collected for analysis from July to September, depending on the ripening stage. For the analyses, 1 kg of fruits was randomly harvested from each block of each treatment. 

**Figure 5:**
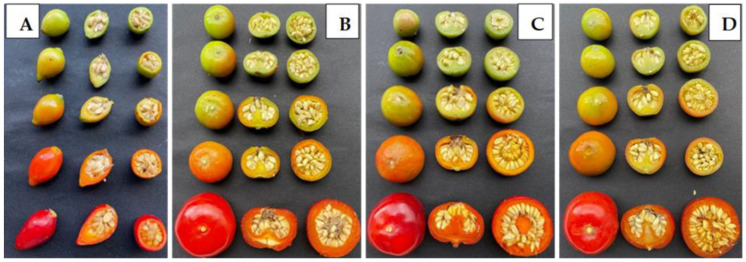
Fruits at ripening stages (I–V) for: (**A**)—*Rosa canina*, (**B**)—*Rosa rugosa*, (**C**)—*Rosa rugosa* ‘Rubra’, (**D**)—*Rosa rugosa* ‘Alba’ (photos by B. Medveckienė).

The rose fruits were harvested at five ripening stages (I-V; Figure 5): (I) was reached when fruit colour changed slightly from green to yellow, pink, or red in no less than 10% of its surface; (II) fruit colour changed from green to tarnish—yellow, pink, or red in no less than 30% of the surface; (III) fruit colour changed from green to light orange or red or a combination thereof in no less than 60% of the surface; (IV) fruit became pinkish or orange depending on the species; (V) fruit surface was red.

Compared to standard climate normal (SCN), in 2018, 2019, and 2020, the temperature was higher by 2.5, 1.4, and 0.7 ℃. During the rosehip vegetation period of 2018, 2019, and 2020, the average precipitation was 86, 106.6, and 72.5 mm less than in the SCN. In addition, in 2018, 2019, and 2020, the sunshine was 195, 118, and 8 h longer than SCN (Table 4).

### 3.2. Soil Agrochemical Analyses 

The soil agrochemical characteristic was evaluated in the Vytautas Magnus University Agriculture Academy Laboratory of Food Raw materials, Agronomical and Zoo-technical Investigations. From the soil samples, the small stones, roots, and other organic plant parts were removed, then air-dried in open plastic boxes and crushed. Homogenised soil was sieved through a 1 mm mesh size sieve. Soil samples were analysed for pH in potassium chloride (KCl) solution (pH_KCl_), plant-available phosphorus, potassium, and total nitrogen. Soil pH_KCl_ was established by the potentiometric method in 1N KCl extract [43]. Plant-available phosphorus and potassium were extracted with ammonium lactate according to the Egner–Riehm–Domingo method [44]. Total nitrogen concentration (mg kg^−1^) was determined by the Kjeldahl method using Kjeldatherm (Gerhardt, Königswinter, Germany) [45].

### 3.3. Pomological Parameters

Fruit length and width in mm were measured with a digital calliper. The fresh fruit biomass (g), flesh and seed weights (g) were measured using a technical balance (Radwag, WPS 510/C/1, Radom, Poland). The results are displayed in grams with a precision of 0.01 g. The fruit-to-flesh ratio was calculated by dividing flesh weight by fruit weight and multiplying by 100. The pomological fruit parameters were calculated as the mean of 60 (3 replicates × 20 fruits) fruits.

### 3.4. Colour Parameter Analysis 

The CIE colour parameters L* (lightness), a* (positive—red, negative—green), and b* (positive—yellow, negative—blue) of rosehips were measured using a spectrophotometer ColorFlex (Hunter Associates Laboratory, Inc., Reston, VA, USA) in NBS units. 

Chroma presents the quality and intensity of fruit colour. Calculations were performed on colour attributes chroma (C), using the following formula:

(1) $$C=\frac{\left(a*\;\cdot \;2\right)+{\left(b*\;\cdot \;2\right)}^{\cdot 1}}{2};$$

Hue angle (h°) was calculated using the following formula:

(2) $$h{}^{\circ}=\arctan\left(\frac{b*}{a*}\right)$$

The colour parameters were calculated as the mean of 60 (3 replicates × 20 fruits) fruits.

### 3.5. Fruit Skin Texture Analysis

The skin elasticity and strength of rosehips were measured using a texture analyser, TA.XTPlus, with a P/2 probe (Stable Micro Systems, Godalming, UK).

The probe descended into the sample at a speed of 1 mm s^−1^ and reached a depth of 5 mm. Skin strength is the force (N) required to puncture the fruit skin, whereas skin elasticity is the distance (mm) to which the skin deflects before probe penetration.

The texture parameters were calculated as the mean of 60 (3 replicates × 20 fruits) fruits.

### 3.6. Statistical Analysis

Data were analysed using Microsoft^®^Excel^®^2016 MSO and confirmed using the STATISTICA 10 (StatSoft, Inc., Tulsa, OK, USA, 2010). All analyses were conducted in triplicate, with the mean and standard deviation presented. Since the analysis of variance did not show year interaction, the data are presented as two-year averages. The samples were defined by two qualitative factors (species/cultivars and ripening stage), and therefore, the reliability of the results was evaluated by a two-way analysis of variance (ANOVA) method. Tukey’s test was applied to assess significant differences between the means at *p* < 0.05. The nature and strength of the correlations between the variables were determined using correlation analysis. Finally, the relationships between rosehip samples of different species/cultivars harvested at five ripening stages and the pomological, colour, and texture properties were evaluated with the XLSTAT 2018 (New York, NY, USA) using principal components analysis (PCA).

## 4. Conclusions

The results of this experimental investigation revealed that the genotype and ripening stage significantly affected the fruit’s pomological, colour, and textural parameters. According to our results, appropriate for transportation was *Rosa canina* having the strongest fruit skin elasticity (3.03 mm) and strength (17.36 N). *R. rugosa*, *R. rugosa* ‘Rubra’, and *R. rugosa* ‘Alba’ are suitable for the industry because these species/cultivars had the largest-sized fruits and the highest flesh ratio at the ripening stage V. Comparing the species and (or) cultivars, *Rosa rugosa* fruits were the reddest, at ripening stage V. The pomological, colour, and texture features of rosehips can be optimised and used for many purposes with the appropriate harvesting time and species/cultivar selection.

## Figures and Tables

**Table 1:** Pomological parameters of rosehip fruits of different species/cultivars during five ripening stages (n = 60).

Species/ Cultivars	Ripening Stage	Pomological Parameters				
		Fruit Width, mm	Fruit Length, mm	Fruit Weight, g	Flesh Weight, g	Seeds Weight, g
*Rosa canina*	I	11.35 ± 0.54 n	16.20 ± 0.51 y	2.33 ± 0.15 i	1.64 ± 0.17 h	0.56 ± 0.08 h
*Rosa rugosa*		19.16 ± 0.45 jk	14.93 ± 0.57 j	3.12 ± 0.18 hi	2.73 ± 0.18 g	0.39 ± 0.05 i
*Rosa rugosa* ‘Rubra’		20.91 ± 0.81 hiy	17.49 ± 0.30 ih	3.44 ± 0.31 gh	2.66 ± 0.34 g	0.79 ± 0.07 efg
*Rosa rugosa* ‘Alba’		18.15 ± 0.54 k	14.96 ± 0.49 j	2.37 ± 0.13 i	1.73 ± 0.13 h	0.63 ± 0.06 h
*Rosa canina*	II	12.33 ± 0.42 n	17.08 ± 0.49 iy	3.02 ± 0.19 hi	2.46 ± 0.21 gh	0.69 ± 0.10 fgh
*Rosa rugosa*		20.76 ± 0.58 iy	16.09 ± 0.63 y	4.22 ± 0.37 fg	3.68 ± 0.39 ef	0.54 ± 0.07 h
*Rosa rugosa* ‘Rubra’		21.86 ± 0.90 gh	18.28 ± 0.60 gh	4.51 ± 0.32 def	3.69 ± 0.31 ef	0.81 ± 0.07 def
*Rosa rugosa* ‘Alba’		20.21 ± 0.47 yj	17.02 ± 0.68 iy	2.96 ± 0.13 hi	2.31 ± 0.34 gh	0.65 ± 0.09 gh
*Rosa canina*	III	13.75 ± 0.54 m	18.71 ± 0.57 fg	4.40 ± 0.52 ef	3.59 ± 0.45 f	0.82 ± 0.11 def
*Rosa rugosa*		23.05 ± 0.52 ef	18.05 ± 0.72 ghi	5.21 ± 0.33 de	4.36 ± 0.25 ef	0.85 ± 0.15 de
*Rosa rugosa* ‘Rubra’		22.88 ± 0.53 fg	19.08 ± 0.65 efg	5.31 ± 0.49 d	4.43 ± 0.50 e	0.88 ± 0.10 de
*Rosa rugosa* ‘Alba’		21.79 ± 0.79 ghi	19.76 ± 0.86 def	4.40 ± 0.52 ef	3.59 ± 0.45 f	0.82 ± 0.10 def
*Rosa canina*	IV	14.64 ± 0.66 lm	21.86 ± 0.64 c	6.97 ± 0.70 c	5.91 ± 0.75 d	1.06 ± 0.10 bc
*Rosa rugosa*		25.91 ± 0.49 c	19.97 ± 0.66 de	7.45 ± 0.39 c	6.51 ± 0.36 d	0.95 ± 0.10 cd
*Rosa rugosa* ‘Rubra’		24.30 ± 0.42 d	20.69 ± 0.61 d	7.36 ± 0.84 c	6.46 ± 0.89 d	0.89 ± 0.09 de
*Rosa rugosa* ‘Alba’		24.00 ± 0.51 de	21.82 ± 0.80 c	7.32 ± 0.98 c	6.42 ± 0.91 d	0.90 ± 0.08 de
*Rosa canina*	V	15.02 ± 0.45 l	24.93 ± 0.82 a	10.22 ± 1.11 b	8.80 ± 0.78 c	1.42 ± 0.15 a
*Rosa rugosa*		31.88 ± 1.03 a	22.91 ± 0.98 c	11.40 ± 0.71 b	10.30 ± 0.69 b	1.10 ± 0.09 b
*Rosa rugosa* ‘Rubra’		29.95 ± 0.96 b	23.80 ± 1.36 b	12.53 ± 0.89 a	11.33 ± 0.68 a	1.19 ± 0.14 b
*Rosa rugosa* ‘Alba’		30.36 ± 1.37 b	22.12 ± 0.41 c	13.79 ± 0.96 a	12.65 ± 0.77 a	1.14 ± 0.10 b

Data are given as the arithmetic mean ± standard deviation with ANOVA *p*-value. Averages in the column followed by different letters are significantly different at 5% probability level (*p* < 0.05). The effects of species/cultivars, ripening stage, and their interaction are significant at *p* < 0.0001.

**Table 2:** Colour parameters of rosehip fruits of different species/cultivars during five ripening stages (n = 60).

Species/ Cultivars	Ripening Stage	Colour Parameters				
		L*	a*	b*	C	h°
*Rosa canina*	I	35.17 ± 0.58 e	−4.52 ± 0.27 j	30.69 ± 1.03 gh	31.03 ± 0.97 i	98.39 ± 0.78 a
*Rosa rugosa*		38.44 ± 0.49 ab	−3.86 ± 0.24 j	35.39 ± 0.76 cd	35.60 ±0.78 g	96.22 ± 0.47 b
*Rosa rugosa* ‘Rubra’		34.71 ± 0.33 e	−3.24 ± 0.18 j	32.89 ± 0.59 ef	33.04 ± 0.58 h	95.63 ± 0.43 bc
*Rosa rugosa* ‘Alba’		39.31 ± 0.38 a	−3.85 ± 0.28 j	35.21 ± 0.62 cd	35.42 ± 0.60 g	96.25 ± 0.53 b
*Rosa canina*	II	37.49 ± 0.34 bc	−2.53 ± 0.26 y	33.26 ± 0.67 ef	33.35 ± 0.67 h	94.34 ± 0.44 bcd
*Rosa rugosa*		37.61 ± 0.67 bc	−2.52 ± 0.32 y	38.62 ± 0.75 b	38.70 ± 0.74 f	93.74 ± 0.50 cd
*Rosa rugosa* ‘Rubra’		36.06 ± 0.53 de	−1.89 ± 0.15 y	34.00 ± 0.50 de	34.05 ± 0.50 gh	93.19 ± 0.24 d
*Rosa rugosa* ‘Alba’		38.56 ± 0.49 ab	−2.16 ± 0.26 y	35.54 ±0.56 cd	35.61 ± 0.55 g	93.48 ± 0.45 d
*Rosa canina*	III	36.03 ± 0.51 de	6.96 ± 0.54 i	31.06 ± 0.67 gh	31.84 ± 0.57 i	77.36 ± 1.16 e
*Rosa rugosa*		39.24 ± 0.26 a	15.96 ± 0.43 g	41.51 ± 0.53 a	44.01 ± 0.65 c	68.74 ± 0.41 g
*Rosa rugosa* ‘Rubra’		37.02 ± 0.15 cd	15.75 ± 0.44 g	35.13 ± 0.64 cd	38.50 ± 0.51 f	65.85 ± 0.89 h
*Rosa rugosa* ‘Alba’		38.17 ± 0.64 abc	12.76 ± 0.92 h	36.79 ± 0.74 c	38.95 ± 0.54 f	70.86 ± 1.55 f
*Rosa canina*	IV	32.84 ± 0.76 f	28.18 ± 1.05 f	29.96 ± 0.77 hi	41.13 ± 1.16 e	46.76 ± 0.80 j
*Rosa rugosa*		38.07 ± 0.70 abc	30.24 ± 0.60 e	39.65 ± 0.51 b	49.87 ± 0.28 a	52.67 ± 0.85 i
*Rosa rugosa* ‘Rubra’		33.27 ± 0.30 f	33.71 ± 0.50 c	32.89 ± 0.57 ef	47.09 ± 0.61 b	44.30 ± 0.53 y
*Rosa rugosa* ‘Alba’		35.23 ± 0.34 e	30.80 ± 0.48 e	31.85 ± 0.76 fg	44.30 ± 0.41 c	45.96 ± 1.03 jy
*Rosa canina*	V	26.60 ± 0.38 h	35.00 ± 0.60 bc	22.53 ± 0.88 j	41.63 ± 0.90 de	32.77 ± 0.82 m
*Rosa rugosa*		36.01 ± 0.63 de	37.83 ± 0.50 a	30.00 ± 0.56 hi	48.28 ± 0.61 a	38.41 ± 0.51 l
*Rosa rugosa* ‘Rubra’		28.92 ± 0.96 g	35.59 ± 0.56 b	26.50 ± 0.49 y	44.37 ± 0.68 c	36.67 ± 0.37 l
*Rosa rugosa* ‘Alba’		29.38 ± 0.52 g	32.25 ± 0.69 d	28.65 ± 0.44 i	43.14 ± 0.50 cd	41.63 ± 0.86 k

Data are given as the arithmetic mean ± standard deviation with ANOVA *p*-value. Averages in the column followed by the different letters are significantly different at the 5% probability level (*p* < 0.05). The effects of species/cultivars, ripening stage, and their interaction are significant at *p* < 0.0001.

**Table 3:** Correlation matrix between pomological, colour, and texture properties.

Traits		1.	2.	3.	4.	5.	6.	7.	8.	9.	10.	11.	12.
1.	Fruit weight	1.000											
2.	Flesh weight	0.999	1.000										
3.	Seeds weight	0.778	0.745	1.000									
4.	Fruit width	0.675	0.685	0.387	1.000								
5.	Fruit length	0.856	0.844	0.792	0.440	1.000							
6.	L*	−0.726	−0.714	−0.698	NS	−0.751	1.000						
7.	a*	0.876	0.870	0.736	0.574	0.910	−0.603	1.000					
8.	b*	−0.565	−0.554	−0.569	NS	−0.641	0.865	−0.442	1.000				
9.	C	0.719	0.721	0.524	0.716	0.659	NS	0.868	NS	1.000			
10.	h◦	−0.885	−0.877	−0.763	−0.531	−0.933	0.657	−0.994	0.504	−0.823	1.000		
11.	Skin elasticity	−0.737	−0.741	−0.518	−0.876	−0.526	NS	−0.633	NS	−0.678	0.603	1.000	
12.	Skin strength	−0.878	−0.880	−0.650	−0.641	−0.800	0.580	−0.855	0.465	−0.699	0.839	0.682	1.000

Significant at *p* < 0.001; NS—not significant.

**Table 4:** Weather conditions during the rosehip vegetation period in 2018, 2019, and 2020 (Šiauliai meteorological station, Lithuania).

Year	Month						
	May	June	June	August	September	Average	
Air temperature, °C							
2018	17.1	17.4	19.6	19.2	14.5	17.6	
2019	13.4	21.2	17.2	18.2	12.5	16.5	
2020	10.4	18.8	17.0	17.9	14.7	15.8	
SCN	12.8	15.7	18.0	17.1	12.0	15.1	
Rainfall, mm							
2018	27.5	16.0	107.9	65.6	57.0	274	
2019	28.6	27.5	50.3	100.5	46.5	253.4	
2020	32.8	106.8	79.3	46.7	21.9	287.5	
SCN	57	73	89	75	66	360	
Sunshine, h							
2018	365	286	210	276	207	1344	
2019	232	349	233	264	189	1267	
2020	260	250	229	222	196	1157	
SCN	252	246	260	237	154	1149	

* SCN—standard climate normal is the 30-year average from 1981 to 2010.
